# Hydrogel-forming microneedle arrays as a therapeutic option for transdermal esketamine delivery

**DOI:** 10.1016/j.jconrel.2020.03.026

**Published:** 2020-06-10

**Authors:** Aaron J. Courtenay, Emma McAlister, Maelíosa T.C. McCrudden, Lalit Vora, Lilach Steiner, Galit Levin, Etgar Levy-Nissenbaum, Nava Shterman, Mary-Carmel Kearney, Helen O. McCarthy, Ryan F. Donnelly

**Affiliations:** a School of Pharmacy, Queen's University Belfast, 97 Lisburn Road, Belfast BT9 7BL, United Kingdom; b School of Pharmacy and Pharmaceutical Sciences, Ulster University, Cromore Road, Coleraine BT52 1SA, United Kingdom; c TEVA Pharmaceuticals, Basel Street 5, Petah Tikvah, Netanya Area, Israel

**Keywords:** Microneedle array, Esketamine, *In vivo*, Treatment resistant depression

## Abstract

Treatment resistant depression is, by definition, difficult to treat using standard therapeutic interventions. Recently, esketamine has been shown as a viable rescue treatment option in patients in depressive crisis states. However, IV administration is associated with a number of drawbacks and advanced delivery platforms could provide an alternative parenteral route of esketamine dosing in patients. Hydrogel-forming microneedle arrays facilitate transdermal delivery of drugs by penetrating the outer layer of the skins surface, absorbing interstitial skin fluid and swelling. This subsequently facilitates permeation of medicines into the dermal microcirculation. This paper outlines the *in vitro* formulation development for hydrogel-forming microneedle arrays containing esketamine. Analytical methods for the detection and quantitation of esketamine were developed and validated according to International Conference on Harmonisation standards. Hydrogel-forming microneedle arrays were fully characterised for their mechanical strength and skin insertion properties. Furthermore, a series of esketamine containing polymeric films and lyophilised reservoirs were assessed as drug reservoir candidates. Dissolution testing and content drug recovery was carried out, followed by permeation studies using 350 μm thick neonatal porcine skin in modified Franz cell apparatus. Lead reservoir candidates were selected based on measured physicochemical properties and brought forward for testing in female Sprague-Dawley rats. Plasma samples were analysed using reverse phase high performance liquid chromatography for esketamine. Both polymeric film and lyophilised reservoirs candidate patches achieved esketamine plasma concentrations higher than the target concentration of 0.15–0.3 μg/ml over 24 h. Mean plasma concentrations in rats, 24 h post-application of microneedle patches with drug reservoir F3 and LW3, were 0.260 μg/ml and 0.498 μg/ml, respectively. This developmental study highlights the potential success of hydrogel-forming microneedle arrays as a transdermal drug delivery platform for ESK and supports moving to *in vivo* tests in a larger animal model.

## Highlights

• Esketamine may be a therapeutic option in treatment-resistant depression.
• Novel drug delivery methods are needed to reduce dosing frequency.
• Microneedle technology has proven benefits as a method of minimally invasive drug delivery.
• Hydrogel-forming microneedles enhance transdermal delivery of esketamine *in vivo.*
• Therapeutic plasma levels of >0.15–0.3 μg/ml were achieved in rats over 24 h.

## Graphical abstract

**Figure:**
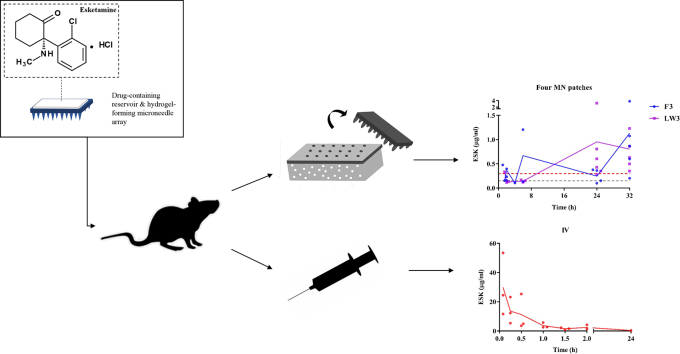

## 1 Introduction

Treatment resistant depression (TRD) affects 2.7 million people in the UK, accounting for between 10 and 30% of people suffering with depression. [1] A recent report by NICE outlined that many patients with TRD have often tried up to 12 antidepressant drugs over 10 years before being referred to specialists. NICE have since updated their guidance stating all patients who have not responded to two antidepressants should be referred to specialists for treatment [1]. Since 1996 it has been known that ketamine and some of its derivatives have an almost immediate effect on TRD with patient's symptoms improved within hours and the effects lasting for several days [2]. Esketamine (ESK) is an anaesthetic agent that has shown rapid antidepressant effects in a number of small clinical studies however there has been much debate over the true clinical effectiveness in TRD [3]. It has been shown to exhibit greater binding affinity to the *N*-methyl-d-aspartate (NMDA) receptor than R-ketamine. This increased affinity has recently been exploited by researchers for its therapeutic applications in TRD. ESK is currently available as intravenous (IV) and intramuscular (IM) injections for human use. Although parenteral injectable drug delivery strategies provide rapid dose delivery they are subject to a number of significant drawbacks. The need for trained personnel to deliver the dose, the use of hypodermic needle and subsequent need for sharps disposal all contribute to increased cost of treatment and potential harm to patients. Similarly, use of hypodermic needles potentiates the risk of blood borne infections and so the development of alternative drug delivery strategies constitutes a current unmet clinical need. In March 2019, the FDA approved Spravato by Janssen, an intranasal ESK spray for TRD after a four-week clinical study showed patient improvement compared with placebo, and oral antidepressants [4]. Although this is a positive step towards developing new formulations for TRD, to ensure all suitable patients can benefit from ESK treatment it is important that alternative routes of delivery are considered.

Hydrogel-forming microneedle (MN) arrays have been shown to facilitate transdermal delivery of a range of small molecule drugs and biotherpaeutic agents [[5], [6], [7]]. The use of MN arrays proposes a number of improvements for ESK delivery that could be useful in TRD. The proposed MN transdermal delivery technology offers a novel approach for enabling continuous delivery of ESK (without pain). Transdermal delivery of the drug promoted by intradermal administration using MNs. ESK is an example of a BCS Class 1 drug with high solubility and high permeability, making it a suitable candidate for hydrogel-forming and dissolving MN technologies. The developed system aims to maintain constant plasma levels that will improve efficacy and compliance for patients. As a parenteral route of administration, bypassing hepatic first pass, reduced metabolism may also be expected.

**Fig. 1:**
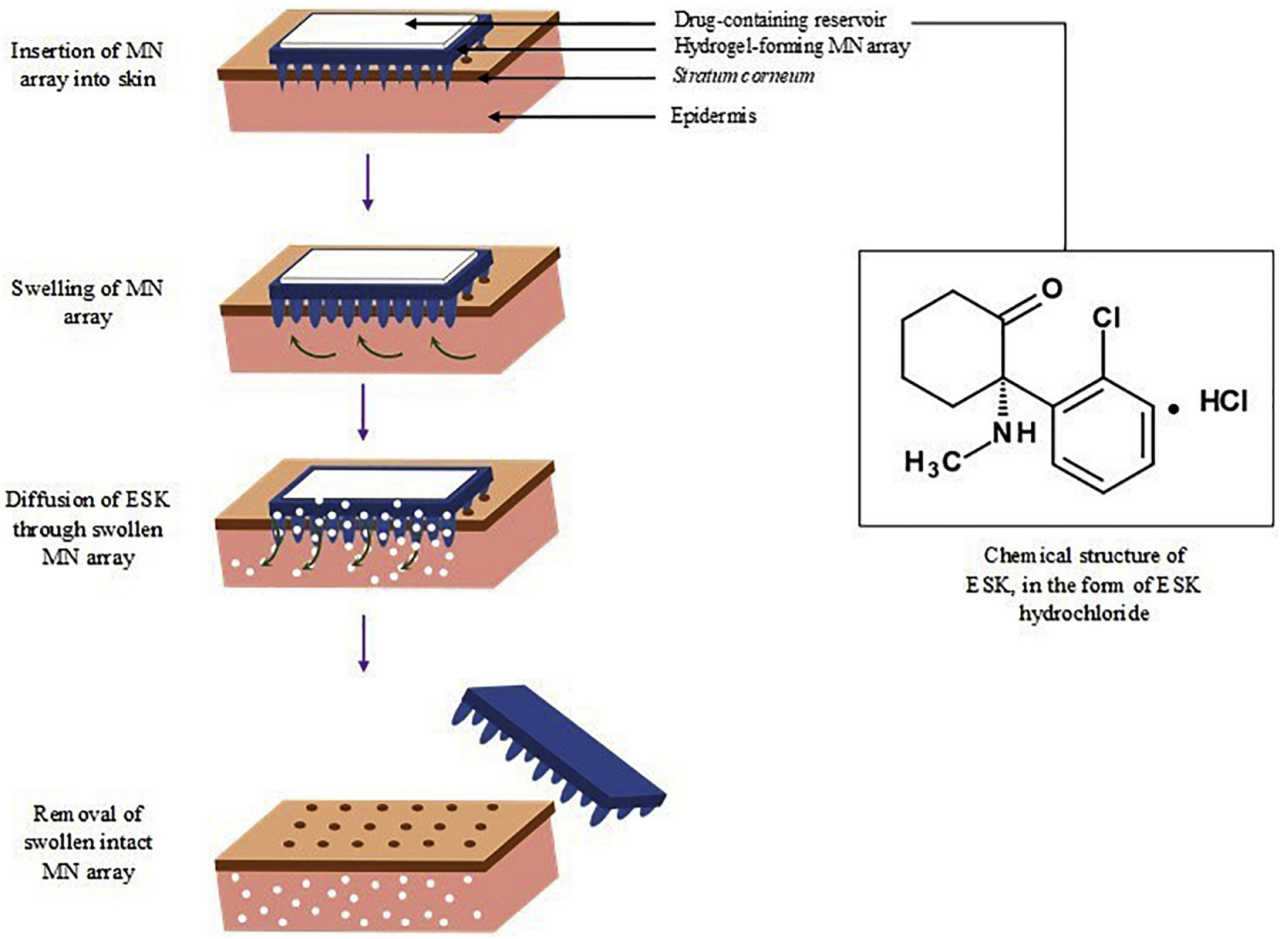
Schematic representation of the mechanism of action of a ESK-containing MN patch. ESK-containing MN patches consist of hydrogel-forming MN arrays and ESK-containing reservoir. Hydrogel-forming MN arrays take up skin interstitial fluid, inducing diffusion of ESK from an ESK-containing reservoir through the swollen micro-projections.

Traditional soluble MN arrays or biodegradable MN arrays refer to those made from dissolving or degradable polymers. Following penetration of the *stratum corneum* (*SC),* the MN arrays containing drug compounds dissolve or degrade within the interstitial fluid held within the dermal microcirculation. The resulting dissolution facilitates drug release. Each of these MN array designs by-pass the *SC* and facilitate delivery of drugs into the dermal microcirculation. However, they are limited by only being able to deliver relatively low doses, often of high potency compounds. The most recent addition to MN technology are hydrogel-forming MN arrays, which are fabricated from polymeric materials that have been crosslinked. The MN arrays pierce the *SC* and draw up interstitial fluid, causing the polymeric matrix to swell. Molecular diffusion of drug substances through the swollen matrix allows for delivery of therapeutic agents into the dermal tissue (Fig. 1). Hydrogel-forming MN arrays contain no drug and, as such, are therefore not limited by the quantity of drug that can be loaded into the needles or onto the needle surfaces. Instead drugs can be loaded into an accompanying reservoir, for example a polymeric film, directly compressed tablet or lyophilised reservoir [6]. This greatly increases the amount of drug that can permeate through the MN array and into the skin.

This study outlines the design and characterisation of hydrogel-forming MN, with particular focus on novel ESK-containing drug reservoir candidates, such as: thin film polymeric formulations and lyophilised reservoirs. A suitable reverse phase high performance liquid chromatography (RP-HPLC) method for separation and detection of ESK from *in vitro* and *in vivo* plasma samples was developed and validated according to International Conference on Harmonisation (ICH) standards and guidance. Initial stability studies of ESK in solution and in candidate formulations are reported, and *in vitro* permeation assessment is carried out using Franz diffusion cell apparatus. Based on the therapeutic concentration of ESK in patients, the aim was to deliver 30–100 mg of ESK over 24 h *in vitro* using Franz Diffusion cell apparatus. Lead candidate ESK-containing reservoirs were selected based on physicochemical analysis and brought forward for testing *in vivo*, in Sprague-Dawley rats. The authors aimed to achieve sustained therapeutic levels of 0.15–0.3 μg/ml in plasma over 24 h using ESK-containing drug reservoirs in combination with hydrogel forming MNs in this *in vivo* feasibility study.

## 2 Materials and methods

### 2.1 Materials

ESK, in the form of ESK hydrochloride (HCl) was purchased from CU Chemie Uetikon, Switzerland. Cryogel SG3 purchased from PB Gelatins, Pontypridd, UK. Pearlitol, 50C-Mannitol was purchased from Roquette, Lestrem, France. Sucrose was purchased from Sigma-Aldrich, Dorset, U.K. Sodium chloride (NaCl) was purchased from Sigma-Aldrich, Steinheim, Germany. Sodium carbonate (Na_2_CO_3_) and Perchloroacetic acid were purchased from Sigma-Aldrich, Steinheim, Germany. Gantrez S-97 was gifted by Ashland Pharmaceutical, Kidderminster, UK. Poly(vinyl alcohol) (PVA) MW 9000–10,000 Da and Tri(propylene glycol) methyl ether (TPME) were purchased from Sigma-Aldrich, Steinheim, Germany. Nair Gentle hair removal cream was purchased from Nair Co., London, U.K. Electric hair clippers were bought from Remmington Co., London, U.K. Franz cell apparatus was purchased from Crown Glass Co. Sommerville, New Jersey, USA. Cyanoacrylate glue was purchased from Loctite Dublin, Ireland. SpeedMixer, DAC 150 FVZ-K, was purchased from Synergy Devices Ltd., U.K. Virtis Advantage Benchtop Freeze- Drier System was purchased from SP Scientific, Warminster PA, USA. The patch occlusive (Scotchpak 9523) was purchased from 3 M Carrickmines, Ireland. The occlusive layer was fixed to the MN patch using an adhesive (DuroTak 87–2100), which was purchased from National Starch and Chemical Company, Bridgewater, New Jersey, USA.

### 2.2 Formulation of hydrogel-forming MN arrays

Hydrogel-forming MN arrays were prepared using laser-engineered silicone micromoulds manufactured, as described previously [8]. The MN arrays were comprised of 121 needles (11 × 11) having a needle height of 600 μm, base width of 300 μm and a base interspacing of 150 μm. The needles were conical shaped and each array had an approximate base area of 0.5 cm^2^. Hydrogel-forming MN arrays, containing no drug themselves were made from aqueous blends of 20% *w*/w Gantrez® S-97, 7.5% w/w PEG 10,000 and 3% w/w anhydrous sodium carbonate. Following this, 0.5 g of the aqueous blend was poured into the moulds, centrifuged at 3000 repetitions per minute (rpm) for 15 min and dried at room temperature for 48 h. Subsequently, the moulds, containing the aqueous blend, were heated at 80 °C for 24 h, facilitating a cross-linking esterification reaction between the carboxylic acid groups of the Gantrez® S-97 and the hydroxyl functional groups of the PEG 10,000 [9]. Upon cooling, the hydrogel-forming MN arrays were removed from the moulds. The hydrogel-forming MN array sidewalls were removed by use of a heated scalpel blade.

### 2.3 Characterisation of hydrogel-forming MN arrays

Parafilm M*®* was used as a model membrane to assess the insertion properties of hydrogel-forming MN arrays into the skin, as described [10]. Briefly, one sheet of Parafilm M*®* was carefully folded such that it formed 8 layers, approximately 1 mm thick. This was then laid onto a poly(ethylene) sheet for support. Hydrogel-forming MN arrays were applied perpendicularly into an eight layer film of Parafilm M® (approximate thickness 1 mm) using a TA.XT.Plus Texture Analyser. In compression mode, the Texture Analyser was programmed to lower at a test speed of 1.19 mm/s and at a force of 32 N for 30 s. After 30 s, the probe was moved upward at a post-test speed of 10.0 mm/s. Texture analyser MN array insertion was compared to manual MN array insertion. Manual insertion studies were conducted by applying thumb pressure to the hydrogel-forming MN array for 30 s into Parafilm M® (prepared as previously described). After 30 s, in both cases, hydrogel-forming MN arrays were removed carefully from the Parafilm M®, the layers of Parafilm M® unfolded and the number of holes in each Parafilm M® layer determined. The percentage of holes in each layer was determined using Eq. (1). From this, an approximate insertion depth was determined.

(1) $$\text{Holes in Parafilm}\;M®\left(\%\right)=\frac{\text{number of holes observed}}{\text{number of holes expected}}*100$$

### 2.4 Preparation and visual assessment of drug-containing reservoirs

A suitable ESK-containing reservoir, intended as a drug-containing reservoir, to be used in conjunction with hydrogel-forming MN arrays was investigated. Firstly, polymeric films containing ESK were produced using a casting method. Polymers were mixed with ESK until a homogeneous blend was obtained. Plasticisers, TPME or PEG 10,000 were added during the preparation stages to improve the flexibility of the polymeric films. The effects of different drug loadings were also investigated. In all cases, concentrations reported for each formulation code refer to initial preparation of aqueous blend made up to 100% *w*/w with water. For each formulation, approximately 30 g of drug-loaded blend was cast into metallic frames (10 × 10 cm). The metallic frame was lined with release liner to ensure the polymeric films could be removed from the frame. The metallic frames were placed on a levelled surface, allowing even spreading of the formulation. The cast blend was then dried at room temperature for 48 h. Following drying, the films were removed from the metallic frames. The summary of the content of formulations investigated for the preparation of ESK polymeric films is presented in Table 1(A).

**Table 1:** (A and B) Summary of the content of formulations for the preparation of ESK-containing (A) films and (B) lyophilised wafers. (C) Lyophilisation parameters for the preparation of ESK-containing lyophilised wafers.

(A)				
Formulation code	ESK (% *w*/w)	Excipients (% w/w)		
		Gantrez® S-97	TPME	PEG 10,000
F1	10	20	10	–
F2	10	20	7.5	–
F3	10	20	5	–
F4	15	20	10	–
F5	15	20	7.5	–
F6	15	20	5	–
F7	20	20	10	–
F8	20	20	7.5	–
F9	20	20	5	–
F10	10	20	–	10
F11	7.5	20	–	10
F12	5	20	–	10


(B)				
Formulation code	Formulation composition (% w/w)			
	ESK	Gelatin	Mannitol	Sodium chloride
LW1	40	10	5	–
LW2	40	10	10	5
LW3	30	10	10	5
LW4	25	10	10	5


(C)	
Temperature (°C)	Time (min)
−40	90
−30	90
−20	90
−10	530
0	30
10	60
25	60

Secondly, ESK lyophilised wafers were prepared. Excipients, such as gelatin, mannitol and sodium chloride were evaluated for their potential use in lyophilised wafers combined with ESK (Table 1(B)). The dry powders of each were weighed and mixed thoroughly using a mortar and pestle. Water was added to the dry powder and mixed in a speed mixer (SpeedMixer™, DAC 150 FVZ-K, Synergy Devices Ltd., UK) at 3000 rpm for 30 s. The resulting ESK formulations (500 mg or 300 mg) were cast into open-ended cylindrical moulds (diameter 13 mm, depth 3 mm), frozen to −80 °C for a minimum 1 h and then placed into a bench-top freeze drier (Virtis Advantage® Bench top Freeze Drier System, SP Scientific, Warminster PA, USA) to be lyophilised. The freeze-drying cycle used is documented in Table 1(C). A vacuum pressure of 600 mTorr was maintained throughout the freeze-drying cycle. In all cases, following lyophilisation, lyophilised wafers were visually inspected for uniformity.

### 2.5 ESK stability in PBS (pH 7.4)

Stability of ESK in PBS (pH 7.4) was assessed. A standard concentration (10 μg/ml) of ESK was prepared in sealed glass vials. In triplicate, these vials were stored at 4 °C, 37 °C, 80 °C, 20 °C (dark) and 20 °C (light). Samples were taken at day 0, 3, 5, 7, 14, 21 and 28 days and assessed using RP-HPLC for the percentage of ESK remaining at that time and condition.

### 2.6 ESK stability in drug-containing reservoirs

Three formulations, Film (F)3, F12 and Lyophilised reservoir (LW)3 were taken forward for further studies. Individual ESK-containing reservoirs of each formulation in triplicate were dissolved in 10 ml PBS (pH 7.4) in glass vials. Following dissolution, the percentage recovery of ESK was determined. Samples were diluted appropriately, filtered and analysed using RP-HPLC.

### 2.7 ESK stability in primary packaging

Stability of ESK in primary packaging was assessed. Primary packaging in this case refers to the inner casing to which MN patches are held. The primary packaging investigated with lead ESK drug-containing reservoirs was 3 M Film Product, Scotchpak™, Minnesota Mining & Manufacturing Co., St. Paul., Minnesota, USA. Each lead formulation, F3 (3 × 0.5 cm^2^), F12 (3 × 0.5 cm^2^) and LW3 (3 × 1 reservoir) were placed into weigh boats and held closed by masking tape. These parcels were then heat sealed (Packer Products, Impulse Heat Sealer P400/C, England, UK) into the primary packaging and stored at ambient conditions (20 °C). At defined intervals, samples were carefully unpackaged, films/reservoirs dissolved in PBS (pH 7.4) (10 ml), and analysed using RP-HPLC.

### 2.8 *In vitro* permeation of ESK from drug-containing reservoirs

The *in vitro* permeation of ESK from MN patches consisting of selected ESK polymeric films of ESK lyophilised wafers in combination with hydrogel-forming MN arrays across dermatomed neonatal porcine skin was investigated. A modified Franz cell diffusion setup was used, which is described previously in Donnelly et al. (2014). Briefly, FDC-400 Franz diffusion cells with flat flange, 15 mm luminal diameter, mounted on a FDCD diffusion drive console providing synchronized stirring at 600 rpm and temperature regulated at 32 ± 1 °C were used. Neonatal porcine skin was acquired from stillborn piglets and excised immediately (<24 h post-partum) and trimmed to 350 μm thickness using an electric Integra Padgett® dermatome Model B (Integra Life Sciences Corporation, Ratingen, Germany). The skin was stored at −20 °C until it was needed. The neonatal porcine skin was shaved and equilibrated in PBS (pH 7.4) for 15 min prior to use. A portion of this skin was secured to the donor compartment of the diffusion cell using cyanoacrylate glue. A hydrogel-forming MN array was applied to the skin using manual application pressure for 30 s. To facilitate adhesion of the polymeric film or lyophilised wafer, 10 μl of water was applied to the back of the MN array. An ESK-containing polymeric film or lyophilised wafer was subsequently placed on top of the MN array. A stainless steel weight (diameter 11 mm, mass 3.5 g) was then placed on top of the ESK-containing polymeric film or lyophilised wafer to help maintain contact between the film or wafer and MN array, and also to ensure MN array insertion throughout the 24 h experiment. The donor cell was secured to the receiver compartment using a stainless steel clamp, and covered with Parafilm M® to reduce evaporation. The receiver compartment contained PBS (pH 7.4), which was degassed prior to use by sonication, Samples were taken (<200 μl) at intervals over the 24 h time period with heat equilibrated PBS (pH 7.4) used to replace sampling fluid The concentrations of ESK in the receiver compartment was quantified using RP-HPLC.

### 2.9 *In vivo* delivery of ESK from Sprague-Dawley rats

Approval for animal experiments was obtained from the committee of the Biological Research Unit, Queen's University Belfast. With the implementation of the principles of the 3Rs (replacement, reduction, and refinement), this *in vivo* experiment was conducted according to the policy of the Federation of European Laboratory Animal Science Associations and the European Convention for the protection of vertebrate animals used for experimental and other scientific purposes.

Female Sprague-Dawley rats (*n* = 18) (Charles River Laboratories, Harlow, UK), were separated into three cohorts (*n* = 6 in the treatment cohorts, *n* = 3 in the control cohort). The transdermal treatments cohorts were rats treated with four MN patches consisting of either formulation code, F3 or LW3 as the drug-containing reservoir. In the control cohort, rats were given ESK solution (5 mg/kg) which was administered intravenously using the lateral caudal tail vein.

For MN patch application, animals were anaesthetised using gas anaesthesia (5% isoflurane inn oxygen, flow 2 l/min). Maintenance anaesthesia was achieved by lowering isoflurane concentration to 2.5% *v*/v, flow 2 l/min. Using electric clippers, the backs of the rats were clipped. Hair removal cream was applied to remove all remaining hairs in the intended application area. Hydrogel-forming MN arrays (4 × MN patches each containing 120 mg ESK) were applied using manual thumb pressure onto a pinched section of skin on the back of the rats for 30 s. The ESK drug-containing reservoir (F3 or LW3) was subsequently placed on top of the MN array and *held in situ* using Microfoam™ surgical tape (3 M, St Paul, Minnesota, USA). Tegaderm™ film was placed over all the MN patches and Micropore™ surgical tape was subsequently wrapped around the back of the rats to hold the MN patches firmly in place for 24 h.

At pre-defined intervals, each rat was heated in a 39 °C heat-box to dilate their tail veins. Using a 23 G hypodermic needle (flushed with heparin solution), blood samples were taken from the rats *via* the tail. In the treatment cohorts, blood samples were collected, at staggered design, at 1.5, 2, 4, 6, 24 and 26 h from 3 animals per time-point. In the control cohort, blood samples were collected at 5, 15, 30 min, 1, 2, 4 and 24 h from 3 animals per time-point. Plasma separation was performed by centrifuging the collected rat blood at 3000 rpm for 10 min at 4 °C in a refrigerated centrifuge. Plasma samples were collected and stored in a freezer at −80 °C until analysis.

All MN patches were removed after 24 h, with adhesive remover spray used to aid removal of the MN patch set up. In all cases, MN patches were visualised and photographed, with comments on the swelling and reservoir dissolution noted.

### 2.10 ESK extraction from Sprague-Dawley rat plasma

Healthy female Sprague-Dawley rats were culled and following cardiac puncture, blood was collected into heparinised micro tubes. This ‘control’ blood was used for assay method development. Plasma separation was performed by centrifuging the blood at 3000 rpm for 10 min in a refrigerated centrifuge (4 °C). The supernatant was extracted, collected and stored at −80 °C until required. To extract ESK from plasma, a number of protein precipitation methods were assessed including addition of acetonitrile however, this yielded inconsistent RP-HPLC traces with many interfering peaks. The optimal protein precipitation agent was perchloroacetic acid. As such, 100 μl of plasma had the protein content precipitated with 100 μl of 0.5 M perchloroacetic acid. The mixture was centrifuged at 15,000 rpm for 15 min at 4 °C. The supernatant was extracted for solid phase extraction (SPE). Oasis HLB Max cartridges were preconditioned with 1 ml of methanol followed by 1 ml of water. The plasma supernatants were added to the cartridge and washed with 600 μl of water. Samples were eluted from SPE cartridges using 1 ml methanol and collected into glass tubes. The methanol was allowed to evaporate at 37 °C for 50 min and the remaining material was reconstituted in 100 μl water. The samples were centrifuged at 13,000 rpm for 15 min at 4 °C. This washing and centrifugation process was repeated in order to remove solid particles and then analysed using RP-HPLC.

### 2.11 Pharmaceutical analysis

A RP-HPLC method was developed to analyse ESK in PBS (pH 7.4) following stability and *in vitro* permeation studies. Using isocratic elution, this method was achieved on an Agilent 1200 series system and Chemstation® computer software B.02.01 was used for chromatogram analysis. The column was a Waters® Xselect® Charged Surface Hybrid C_18_ column (130 Å pore size, 150 mm length × 3.0 mm internal diameter, 3.5 μm particle size) with the temperature of the column maintained at 25 °C. The mobile phase was 0.02 M potassium dihydrogen phosphate (pH 8.0) and methanol in the ratio 70:30% *v*/v with a flow rate of 0.5 ml/min. The injection volume was 20 μl and the UV detector was fixed at 214 nm. The sample run time was 7 min. Standard samples in triplicate of ESK (0.625–20 μg/ml) were prepared in PBS (pH 7.4).

To analyse ESK in rat plasma samples, this RP-HPLC method was modified slightly. The column, column thermostat, mobile phase and UV wavelength parameters remained the same. The flow rate was decreased to 0.35 ml/min, the injection volume was increased to 50 μl and the sample run time was increased to 10 min.

The RP-HPLC methods developed for the detection and quantification of ESK in PBS (pH 7.4) and rat plasma were validated in accordance to the International Conference on Harmonisation (ICH) guidelines (8). Parameters considered during method validation were specificity, linearity, range, accuracy, precision, limit of detection (LoD) and limit of quantification (LoQ). In each method, all the calibration plots were subsequently collated to generate one representative calibration curve for each analytical method. Least squares linear regression analysis and correlation analysis was performed. The LoD and LoQ were determined using the standard deviation (S.D.) of the response and slope of the calibration curve, as described in ICH guidelines.

### 2.12 Pharmacokinetic analysis

Pharmacokinetic (PK) parameters for ESK were calculated using group mean concentration-time data, according to nominal time, by non-compartmental method using Phoenix WinNonlin 6.3. For IV treated group, an intravascular model was used and for MN treated group an extravascular model was used for the analysis. For descriptive statistics, individual plasma concentration below limit of quantitation (BLQ) values were treated as zero. For PK parameters calculation, BLQ values at a sampling time between 2 quantifiable concentrations were treated as zero for calculation and representation purposes. No other BLQ values were observed.

The maximum observed plasma concentration (C_max_) and time to reach C_max_ (t_max_) were obtained directly from the concentration-time data. Terminal elimination half-life (t_1/2_) was calculated as ln(2)/λz. Area under the plasma concentration-*versus*-time curve from time 0 to infinity (AUC_0-∞_) or from time 0 to the last quantifiable time-point (AUC_0-t_) was calculated by means of linear up-logarithmic down trapezoidal summation. AUC_inf_, t_1/2_ as well as CL and Vss were reported as reliable only if terminal elimination phase was adequately characterised: terminal elimination phase includes at least 3 non-BLQ data points after C_max_, adjusted r^2^ value ≥0.85 and AUC_0-*t*_ ≥ 80% AUC_inf_, interval over which terminal elimination slope (λ) is estimated ≥1.5× t_1/2_.

### 2.13 Statistical analysis

All data were expressed as mean ± S.D. Least squares linear regression analysis, correlation analysis, LoD and LoQ were all performed using Microsoft® Excel 2007 (Microsoft Corporation, Redmond, USA). Statistical analysis was performed using GraphPad Prism® version 7 (GraphPad Software, San Diego, USA) and included calculation of mean, standard deviation, construction of calibration plot with least-squares linear regression analysis, and analysis of residuals. Mann–Whitney U, ANOVA, and Student's *t*-test were used as appropriate to assess statistical significance throughout. In all cases, *p* < .05 denoted significance.

## 3 Results

### 3.1 Formulation and characterisation of hydrogel-forming MN arrays

Hydrogel-forming MN arrays were fabricated and demoulded. MN arrays were placed in PBS (pH 7.4) and percentage swelling was recorded through noting the increase in mass. The percentage swelling of hydrogel-forming MN arrays increased to 1760% of their original size at 24 h.

**Fig. 2:**
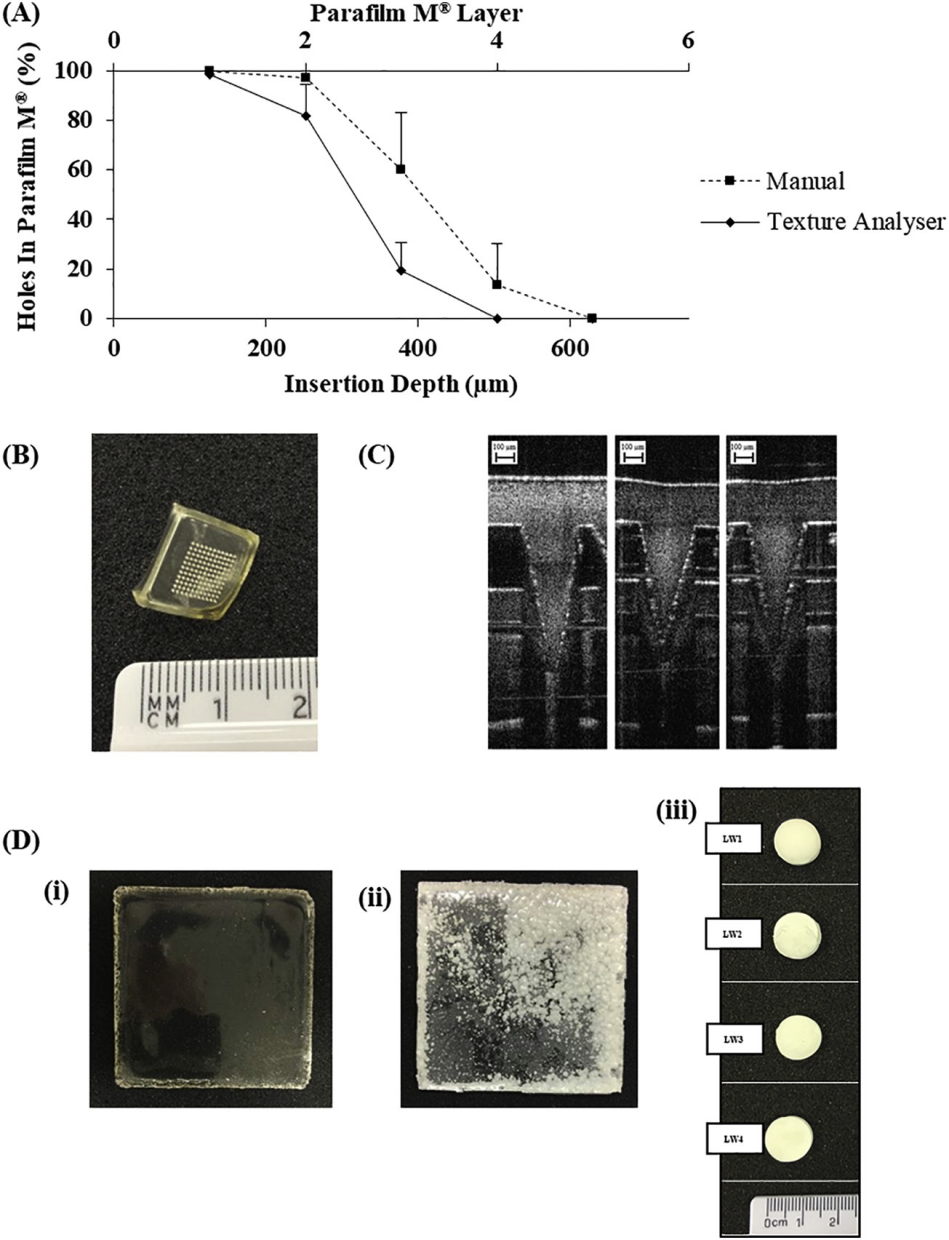
(A) Number of holes created in each Parafilm M® layer expressed as a percentage to the number of holes expected and approximate insertion depth following insertion of hydrogel-forming MN arrays using the Texture Analyser and manual pressure (Means + S.D., *n* = 5). (B) Digital image of MN array. (C) Exemplar optical coherence tomography images of hydrogel-forming MN arrays inserted into Parafilm M® (C) Exemplar digital images of polymeric films and lyophilised wafers prepared. Formulation code (i) F3; (ii) F6 and (iii) LW1, LW2, LW3 and LW4. All digital images were taken with a digital camera.

Hydrogel-forming MN arrays were fabricated and tested for mechanical strength. In this study, following insertion of the MN into Parafilm M, 100% of the needles penetrated the top layer, with 98.1 ± 1.9% of needles under manual pressure and 81.8 ± 12.8% under texture analyser pressure, penetrating to the second layer. This model is consistent with previous reports of hydrogel-forming MN manufactured in this way [11]. Fig. 2B shows exemplar Optical Coherence Tomography images of MNs penetrating into Parafilm M layers.

Compression testing was also carried out to ensure MN tips were mechanically robust enough to withstand application to the skin. Hydrogel-forming MN tip heights were visually assed using a Leica light microscope and found to be 503.8 ± 5.3 μm and 498.1 ± 3.5 μm before and after insertion respectively, indicating good mechanical strength from the tested formulations.

### 3.2 Preparation and visual assessment of drug-containing reservoirs

A number of iterations of ESK-containing reservoirs were formulated in order to optimise the amount of ESK contained within each unit or cm^2^. Each candidate formulation was required to exhibit a number of criteria before more in-depth ESK recovery studies were undertaken. These are: each thin film formulation had to be successfully freed from the mould into which it was cast, flexible enough to be handled from frame to accompanying MN array, and not brittle. In each case a visual inspection was carried out to asses each film formulation for signs of precipitation. Precipitation was defined as any crystalline deposit visible within the formulation. Fig. 2C(i-ii) shows exemplar photographic images of thin film formulation which have resulted on ESK precipitation post drying. Any film formulation that displayed precipitation over the course of the study was rejected and not taken forward as an optimised ESK-containing reservoir candidate.

Lyophilised wafers were visually inspected for uniformity following removal from their plastic moulds, shown in Fig. 2C(iii). Lyophilised reservoir dissolution time was recorded and varied from 6.7 ± 0.9 min to 15.7 ± 1.7 min. Due to the rapid swelling of hydrogel-forming MN it was decided that a short dissolution time of <10 min was appropriate for this dosage form and as such, lead formulations were chosen on these two criteria, dissolution time, and visual uniformity.

### 3.3 Pharmaceutical analysis

The reservoirs were 13 mm in diameter with a thickness of 2.5 mm. The total surface area of the cylindrical reservoirs was 1.32 cm^2^. Percentage recovery of ESK from LR1 and LR3 was high (86.9 ± 6.7% and 87.4 ± 6.2 respectively) and dissolution times were short (15.7 ± 1.7 min and 6.7 ± 0.9 respectively). ESK validation parameters are shown in Table 2 below.

**Table 2:** Validation parameters for RP-HPLC analytical methods, ESK in PBS (pH 7.4) and ESK in rat plasma (Means ± S.D., *n* = 5).

	Analytical method	
	ESK in PBS (pH 7.4)	ESK in rat plasma
Range (μg/ml)	0.625–20	0.2–5
Slope	83.23	0.18
y-intercept	16.89	40.63
r^2^	0.9999	0.9989
LoD (μg/ml)	0.10	0.05
LoQ (μg/ml)	0.30	0.07

### 3.4 ESK stability

**Fig. 3:**
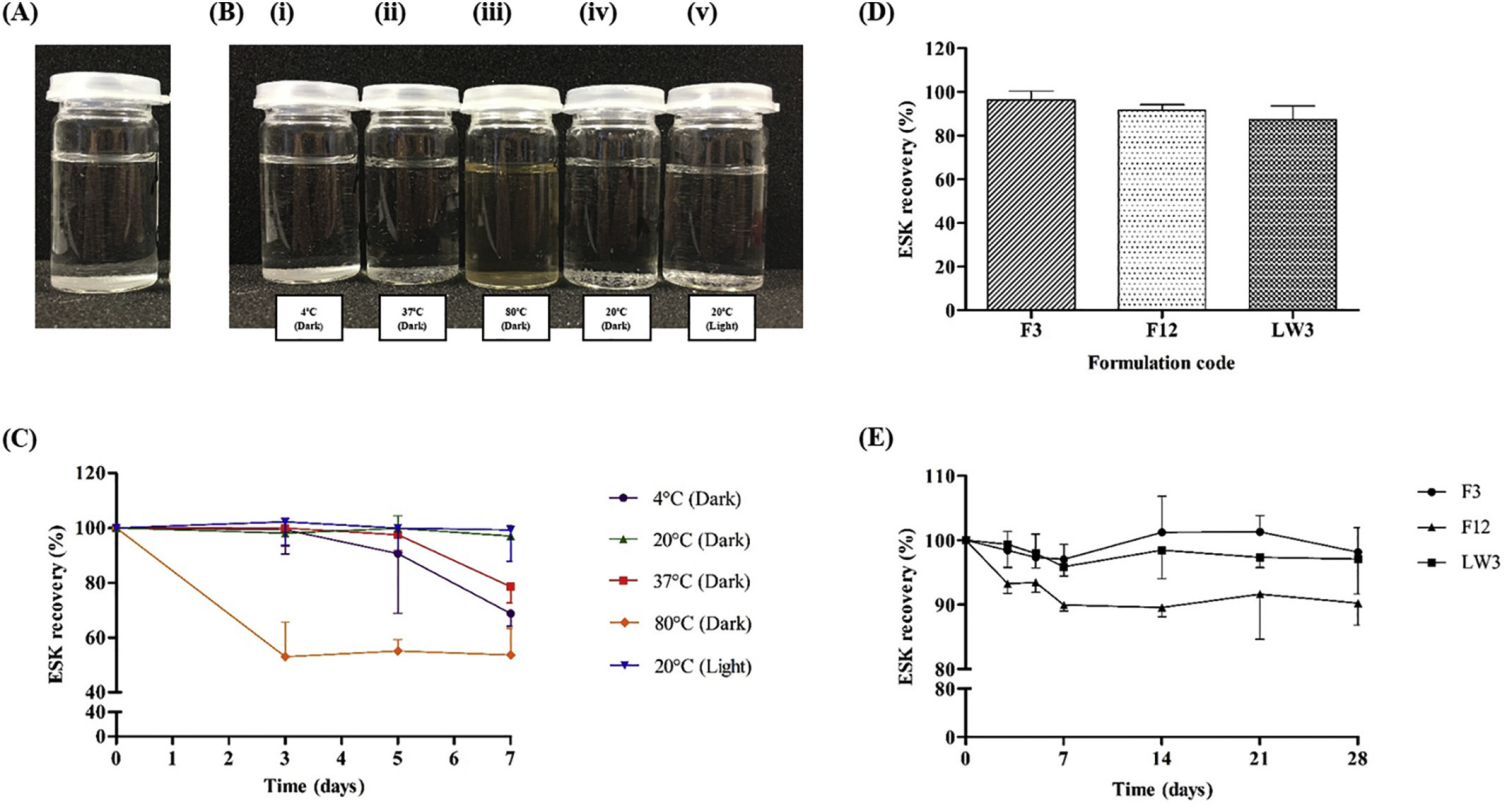
Photographic images of ESK in PBS (pH 7.4) at (A) the beginning of the study and (B) following storage for 3 days under various conditions (i) 4 °C (Dark); (ii) 37 °C (Dark); (iii) 80 °C (Dark); (iv) 20 °C (Dark) and (v) 20 °C (Light). (C) ESK recovery (%) in PBS (pH 7.4) under various conditions up to 7 days (Means ± S.D., *n* = 3). (D) ESK recovery (%) from lead formulations (formulation codes, F3, F12 and LW3) (Means ± S.D., *n* = 3). (E) ESK recovery (%) from lead formulations placed into primary packaging, stored at 20 °C and tested over 28 days (Means ± S.D., *n* = 3).

ESK was visually assessed for degradation and photographic images of ESK in PBS under various storage condition can be seen in Fig. 3A and B. The percentage recovery of ESK from PBS was assessed over seven days as shown in Fig. 3C. ESK was stable with high percentage recovery values recorded up to 3 days, except in the case of storage at elevated temperatures of 80 °C. Fig. 3D shows that at day 7 ESK recovery from F3 was 97.0 ± 2.3%, F12 recovery was 89.9 ± 0.9% and LW3 recovery was 95.9 ± 1.4%. Fig. 3E shows that high percentage recovery values were still reported at 28 days F3 recovery was 98.2%, F12 was 90.3% and LW3 was 97.1% when stored in the test primary packaging.

### 3.5 *In vitro* permeation of ESK from MN patches

In each experimental set-up detectable quantities of ESK were observed in the receiver compartment of the Franz diffusion cell apparatus from the 15 min time point onwards, however appreciable levels of ESK were not seen until the 90 min time point. At 24 h (1440 min) LW3 showed cumulative permeation of 31.9 ± 5.1 mg, F12 cumulative permeation of 5.7 ± 1.0 mg and F3 cumulative permeation of 16.8 ± 2.9 mg. Permeation from each reservoir over the first 6 h showed a similar profile, although F12 had a lesser extent of ESK permeation. Furthermore, within the time period between 6 and 24 h significant permeation of ESK was observed, particularly with LW3.

Looking at the cumulative percentage permeation, it can be seen that although LW3 delivered the highest quantity of ESK this was in fact the lowest percentage quantity of ESK in the formulation. At 24 h the percentage permeation of LW3 was 21.3 ± 3.4%, F12 was 32.1 ± 4.9%, and F3 was 40.1 ± 8.0%.

### 3.6 *In vivo* delivery of ESK from Sprague-Dawley rats

Initially, a pilot study was conducted to ensure the rats were not subject to adverse effects and further to ensure ESK could be detected and quantified using the plasma extraction and HPLC qualified methods. Three females SD rats received a single ESK dose either as 5 mg/kg IV as positive control, or 2 × MN topped by either film (F3) or lyophilized reservoir (LW3), at 30 mg/cm^2^ (total patch size 1 cm^2^) for 24 h and 120 mg/cm^2^ (total patch size 1 cm^2^) for 24 h, respectively. The MN array insertion was confirmed visually upon removal of the patch at *t* = 24 h. Following removal of the MN arrays it was clear that the needles had swollen and the ESK-containing reservoirs (F3/LW3) had dissolved. Visual inspection of the patches upon removal showed that the lyophilised reservoir had dissolved to a greater extent than the film reservoir. In the majority of LW3 a white residual solid (undissolved reservoir) could be observed.

None of the 3 rats showed any signs of ill health or adverse effect through the course of the pilot study period neither as a result of MN insertion nor ESK administration. Unfortunately, ESK was below the limit of detection (50 ng/ml) in the initial sampling points for animals treated with MN + film (F3) and MN + lyophilised reservoir (LW3) of 2 and 4 h, and could only be quantified at the third and last time-point of 24 h post application. At 24 h post application, the plasma concentration at 24 h was well within and above the product target concentration range of 0.15–0.3 μg/ml: 0.2601 μg/ml for F3 and 0.498 μg/ml for LW3.

**Fig. 4:**
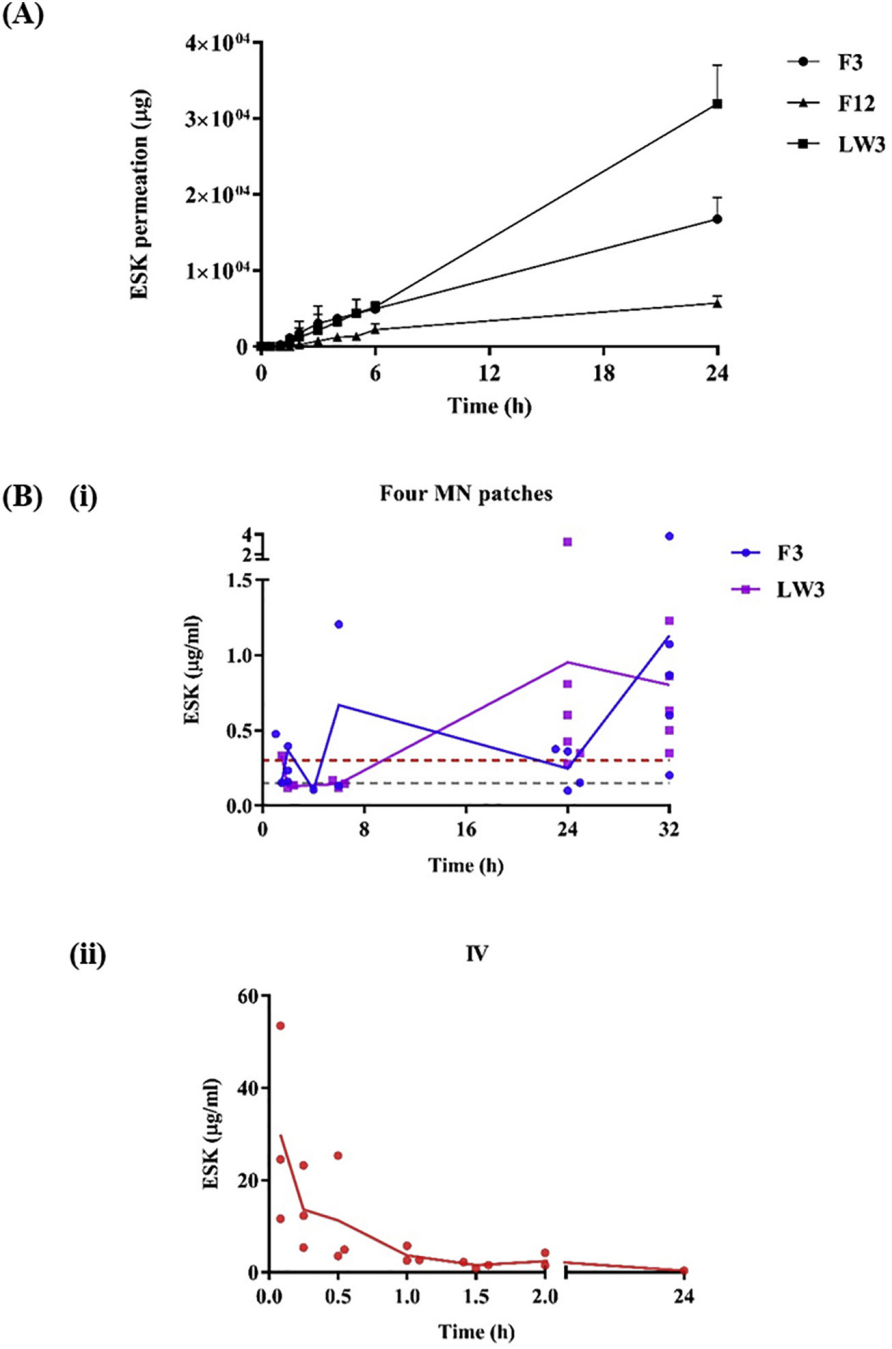
(A) *In vitro* permeation profiles of ESK from MN patches consisting of lead formulations in combination with hydrogel-forming MN arrays (Means + S.D., *n* = 4). (B) ESK concentration in rat plasma. (i) Treatment cohorts that received 4 MN patches consisting of either a film (formulation code, F3) or lyophilised wafer (formulation code, LW3) as the drug-containing reservoir (F3, Means ± S.D., *n* = 3 at 1.5 h, 2 h, 4 h and 6 h; *n* = 4 at 24 h; *n* = 6 at 32 h) (LW3, Means ± S.D., *n* = 3 at 1.5 h, 2 h and 6 h; *n* = 6 at 24 h and 32 h). Red and grey dashed lines indicate the target plasma concentrations, 0.15 μg/ml and 0.3 μg/ml, respectively. Purple and blue solid lines indicate the average ESK concentration in rat plasma. (ii) Control cohort that received ESK solution *via* IV (Means ± S.D., *n* = 3). The red solid line indicates the average ESK concentration in rat plasma. (For interpretation of the references to colour in this figure legend, the reader is referred to the web version of this article.)

In frame of the pivotal study, none of the treated rats showed any signs of ill health or adverse effect through the course of the study period neither as a result of MN insertion nor ESK administration. The IV control cohort, having received ESK 5 mg/kg, displayed characteristic IV ESK plasma concentration-time profile, with multiphasic profile, composed of high initial plasma values with C_0_ back extrapolated to 36.574 μg/ml and rapid initial decline till 1.5 h postdose (beta phase) with t_1/2, beta_ of 0.38 h followed by a slower terminal declined (z phase) with t_1/2,z_ of 9.5 h. AUC_inf_ was 38.686 μg*h/ml and effective (beta phase) clearance (CL) and volume of distribution (V_ss_) were 129 ml/h/kg and 457 ml/kg, respectively (Fig. 4B(ii)).

ESK was quantifiable in all time points through the 1.5 to 26 h post application assessment period. Considering the low sample size and resulting high inter-individual variability, plasma ESK levels for both MN groups were rather stable for the entire observation period, and around or higher than 0.15 μg/ml. The cohort that received F3 showed ESK initial mean concentrations at 1.5 h of 0.086 μg/ml rising to a mean concentration of 0.93 μg/ml at 26 h which was also highest observed concentration (C_max_) for that group. The cohort that received LW3 had a mean initial 1.5 h plasma concentration of ESK of 0.092 μg/ml at 26 h, with C_max_ of 0.789 μg/ml observed at 24 h post application. Fig. 4B(i).

Plasma area under the concentration-time curve from time zero to the last measured time-point, 26 h post-dose was similar for the 2 MN groups with 9.068 μg*h/ml and 9.977 μg*h/ml for F3 and LW3 treated rats. Since over the 26 h observation period terminal elimination phase could not be characterised for the 2 MN groups, further PK characterisation including evaluation of ESK bioavailability through MN administration was not possible.

## 4 Discussion

This work outlines novel combinations of hydrogel-forming MN technology with ESK-containing candidate drug reservoirs. Hydrogel-forming MN technology has been used to deliver a range of therapeutic agents including small molecule drugs, low molecular weight protein compounds, and large antibody therapeutics [[5], [6], [7]]. In the first instance, hydrogel-forming MNs share many of the physical characteristics to that of other MN technologies, including strong, sharp, needle-like projections fabricated on a supporting baseplate. Once applied to the skin however, the hydrogel-forming MN arrays take up interstitial skin fluid and swell. The open and hydrate polymeric network allows for the delivery of a range of therapeutic drugs and compounds to permeate across the skin barrier and into the dermal circulation. One of the main advantages of hydrogel-forming MN technology is that it can facilitate a range of drug delivery profiles that can be tailored. For example, depending on polymeric selection and cross-linking process a rapid high dose release, or slow controlled dose release can be achieved [9]. Furthermore, a transdermal delivery method of ESK may help to prevent potential abuse and misuse of controlled substance drugs as compared to other routes of delivery such as ESK delivery as a nasal spray. Some formulations are prepared in such a way as the drug is difficult to extract by dissolution or with household chemicals, such as bleach, lemon juice, bicaronate of soda solution or vinegar. An acknowledged clinical requirement is to ensure patient safety including measures that would reduce abuse or tampering risk, in line with all other controlled drug preparation risk assessments.

An initial objective of this work was to achieve therapeutic concentrations of permeated ESK *in vitro* using a Franz diffusion cell apparatus. To achieve this, candidate ESK containing drug reservoirs were formulated, such that they were suitable to combine with hydrogel-forming MN arrays. The candidate ESK-containing reservoirs were assessed visually for mechanical robustness, and assayed using RP-HPLC for ESK percentage recovery.

Insertion analysis of MN arrays remains an important aspect of *in vitro* characterisation. If MN arrays do not penetrate the skin, then interstitial skin fluid cannot be imbibed into the hydrogel and drug permeation across the hydrogel network will not occur – rendering the device useless. MN insertion in skin can be visualised using Optical Coherence Tomography, however, it is unlikely that regulators of MN technology will want to rely on the use of biological tissues in quality control assessment techniques. As such, a widely used material, namely Parafilm M, has been developed as a substitute model for MN insertion [11]. Fig. 2B shows the depth of insertion of MN arrays into Parafilm M layers following manual application. In each case, MN insertion is deeper than 200 μm. This is important as the *stratum cornium* is ~50 μm thick, and being the primary barrier to drug diffusion the MN arrays developed here clearly penetrate more deeply and therefore provide a suitable platform for drug delivery.

In order to incorporate ESK into a MN device it was important to understand the stability of ESK *in vitro*. Therefore, a short stability study of ESK in PBS (pH 7.4), the media used in Franz diffusion cell apparatus, was undertaken. The results show that ESK was stable in PBS (pH 7.4) for up to three days, allowing sufficient time for HPLC analysis of ESK before significant degradation occurred. HPLC analysis was carried out immediately after sample processing and within 3 days of experimentation.

The lead film formulations were F3 and F12, as these formulations did not demonstrate recrystallisation or precipitation of ESK, provided sufficient flexibility for ease of handling and importantly had good recovery of the active compound 93 ± 1.4% and 91 ± 2.1%, respectively. Although polymeric formulations are simple to manufacture and can easily be translated to industrial settings, they are limited as drug containing reservoirs by the inherent solubility of the drug in polymer gel formulations. In this case, a balance between the amount of ESK that could be loaded into the films with the resultant brittleness had to be achieved. In many cases, plasticisers were added to improve the flexibility of these films. Recrystallisation and precipitation of ESK from the films on drying proved to be the limiting factor, however F3 and F12 showed promise with regards to ESK loading and a suitable degree of flexibility for handling and patch production.

Lyophilised reservoirs containing ESK were chosen for their porous and hygroscopic nature ensuring that they will readily dissolve in a small quantity of fluid to assist the permeation of drugs through the hydrogel-network [5]. In order to achieve uniform lyophilised reservoirs with an appropriately porous structure mannitol was used as hygroscopic agent and bulking agent. Structural integrity was achieved by use of gelatin and sodium chloride. Uniform lyophilised reservoirs were produced following the lyophilisation process. This indicated that lyophilisation was a conservative method for the preparation of ESK containing reservoirs and further indicated that the reservoirs would readily dissolve on contact with PBS or water. Again the authors selected manufacturing processes for which industrial scale equivalence is already available thus enhancing the potential impact of this work.

Consideration to the primary packaging was given, specifically in terms of maintenance of structural integrity. In the future, MN technology may provide individually packed MN arrays with separately packaged drug containing layers. This would significantly reduce the concerns of stability and packaging throughout the manufacturing process. Hydrogel-forming MN arrays, similar to lyophilised reservoirs, are inherently sensitive to increased levels of moisture, therefore it is important to not only structurally protect these components but further ensure they are protected from humidity. To achieve this, a moisture impermeable packaging was used to envelop the weigh boats containing the reservoirs or MN. In all cases the packaging provided an ideal environment, maintaining the integrity of each MN component. MN arrays were able to penetrate the skin, even after 28 days, and furthermore, ESK was recovered at 28 days F3 98.2%, F12 90.3% and LW3 97.1%. The primary packaging therefore served its main purpose in protecting the materials from increased moisture.

MN arrays facilitated delivery of ESK across dermatomed neonatal porcine skin in each experimental set-up, from every reservoir type (film or lyophilised). It is worth noting that although the highest quantity of ESK delivered across the skin *in vitro* was from the lyophilised reservoir LW3, due to the higher initial loading of ESK this correlated to the lowest efficiency (21.3 ± 3.4%), compared to film formulations F3 and F12 which delivered less ESK but a higher proportion of their initial loading. The ESK concentrations in the receiver compartment of the Franz diffusion cell apparatus steadily increases through 24 h and so this suggests that these patch systems have not exhausted their ESK reserves. This therefore could indicate potential for sustained delivery of ESK for >24 h. A permeation plateau would have been expected at the point where minimal further drug permeation is taking place and as this has not been reached within the 24 h period, there could be the potential for extended treatment times or alternatively higher exposure to ESK *in vivo*.

A pilot study was initiated using 3 rats to ensure no adverse effects, signs of toxicity, or general ill health as a result of ESK delivery of MN application were seen. All animals appeared healthy throughout the course of the study and no adverse reactions were observed. Rat 1 which received IV control administration provided a clear demonstration of high to low plasma concentrations of ESK. The remaining 2 rats treated with MN patches only showed detectable ESK concentrations at 24 h.

MN applications were visually confirmed at the time of application, ensuring the highest chances of success with each patch applied firmly and retained using a secure adhesive system. In each case the MN patches were held securely in place using a combination of Microfoam™ tape, Tegaderm™ dressing, and Micropore™ tape. It was clear that placement of the MN arrays on the rat backs was of high importance. MN that were placed close to the shoulder and hip flexure points were more likely to become loose over the course of 24 h and subsequently be expelled from the skin, leading to patch failure. Fig. 4B(ii) clearly show successful IV administration of ESK in all test animals with relatively small error bars indicating high reproducibility within the cohort. Fig. 4B(i) shows that the film formulation (F3) was able to achieve the target plasma concentration. Following removal of the patches it was apparent that not all of the film formulations had completely dissolved. This was a phenomenon that was also seen with the LW3 lyophilised reservoirs, in fact, the lyophilised reservoirs had only partially dissolved in some cases. The plasma profiles indicated in Fig. 4B(i) a certain degree of fluctuation. This may be as a result of the staggered study design necessitated by Project License limitations. The full pilot study achieved the main aim of ESK plasma concentrations of minimum 0.15–0.3 μg/ml sustained over 24 h. Further, although not within the scope of this project, it is possible that the MN patches had not fully depleted in ESK reserves. It is recommended that future *in vivo* studies should consider application of test MN patches for longer than 24 h to assess the potential for longer term delivery from hydrogel-forming MN patches and also evaluate full pharmacokinetic profiles in order to estimate ESK bioavailability through MN delivery, to elucidate its full exposure potential. In this study, although the MN patches were removed at 24 h time point, the plasma ESK concentrations continue to increase for the F3 film MN patches and are maintained at the 30 h time point for the LW3 MN patches. This suggests that once the ESK has permeated into the skin it is retained in the skin before being slowly released into the systemic circulation. Further research is required to fully understand the pharmacokinetic profile of ESK *in vivo* following transdermal delivery using MN arrays.

It is clear that the ESK plasma profiles in MN and IV cohorts are very different with IV dosing indicating the need for regular repeat administration for patients to achieve long term therapeutic concentrations. As demonstrated in Fig. 4, MN technology can provide sustained ESK delivery, here over 24 h, in rats at relevant and equivalent therapeutic concentrations. The main point of note here is that MN patches can be scaled in size to help tailor dosing for patients and achieve tighter controls on ESK delivery and therefore circulating plasma concentrations.

To advance patient care, it is important to provide new treatment options that patients can use easily and safely, that provide therapeutic effect, and minimal impact on a patient's day to day life. Administration of IV medicines is associated with significant resource burdens for healthcare economies and as such, development of new alternative delivery routes is essential. The next step in development of an ESK delivery system for TRD is to test in larger animal models. For the first time, we outline the development, *in vitro* and *in vivo* assessment of hydrogel-forming MN technology for the delivery of ESK for TRD. This work supports the development of MN technology for transdermal delivery of ESK as a potential method to circumvent first pass metabolism and achieve rapid dosing in patients. Convenient systems such as this will ensure patients receive maximum therapeutic benefit and could contribute to improved healthcare outcomes.

## 5 Conclusion

Hydrogel-forming MN arrays provide an ideal opportunity for enhanced and sustained transdermal delivery of ESK into systemic circulation as a potential therapeutic alternative for patients suffering from TRD. The authors achieved their primary aims by preparing a number polymeric films and lyophilised reservoirs, which underwent rigorous characterisation. Both formulation strategies displayed promise with lead formulations optimised and selected early in the process. *In vitro* assessment was carried out on reservoirs and hydrogel-forming MN arrays. *In vitro* permeation experiments were completed using neonatal porcine skin in Franz diffusion cell apparatus. In parallel to this, primary packaging was developed to facilitate transport and delivery of prototype MN devices. Stability studies of the active compound ESK and mechanical characterisation of MN arrays was assessed over 28-day period and suitable moisture impermeable packaging was selected. *In vivo* assessment of MN-mediated transdermal delivery of ESK was assessed in Sprague Dawley rats with whole blood samples taken, plasma extracted and ESK quantified using qualified HPLC methods. Hydrogel-forming MN arrays provided sustained delivery of ESK in rats at >0.15–0.3 μg/ml plasma concentrations over 24 h. Furthermore, it was clear that the ESK-containing MN patches had not fully exhausted ESK reserves and so displayed the potential for successful use in applications >24 h. Further research is required to fully understand the pharmacokinetic profile ESK *in vivo* following transdermal delivery using MN arrays.

## Declaration of Competing Interest

The authors declare no conflict of interest.
